# MET Gene Amplification and MET Receptor Activation Are Not Sufficient to Predict Efficacy of Combined MET and EGFR Inhibitors in EGFR TKI-Resistant NSCLC Cells

**DOI:** 10.1371/journal.pone.0143333

**Published:** 2015-11-18

**Authors:** Dario Presutti, Simonetta Santini, Beatrice Cardinali, Giuliana Papoff, Cristiana Lalli, Simone Samperna, Valentina Fustaino, Giuseppe Giannini, Giovina Ruberti

**Affiliations:** 1 Institute of Cell Biology and Neurobiology, National Research Council, Campus Adriano Buzzati-Traverso, Monterotondo (Roma), Italy; 2 Department of Experimental Medicine, University La Sapienza, Roma, Italy; Seoul National University, REPUBLIC OF KOREA

## Abstract

Epidermal growth factor receptor (EGFR), member of the human epidermal growth factor receptor (HER) family, plays a critical role in regulating multiple cellular processes including proliferation, differentiation, cell migration and cell survival. Deregulation of the EGFR signaling has been found to be associated with the development of a variety of human malignancies including lung, breast, and ovarian cancers, making inhibition of EGFR the most promising molecular targeted therapy developed in the past decade against cancer. Human non small cell lung cancers (NSCLC) with activating mutations in the *EGFR* gene frequently experience significant tumor regression when treated with EGFR tyrosine kinase inhibitors (TKIs), although acquired resistance invariably develops. Resistance to TKI treatments has been associated to secondary mutations in the *EGFR* gene or to activation of additional bypass signaling pathways including the ones mediated by receptor tyrosine kinases, Fas receptor and NF-kB. In more than 30–40% of cases, however, the mechanisms underpinning drug-resistance are still unknown. The establishment of cellular and mouse models can facilitate the unveiling of mechanisms leading to drug-resistance and the development or validation of novel therapeutic strategies aimed at overcoming resistance and enhancing outcomes in NSCLC patients. Here we describe the establishment and characterization of EGFR TKI-resistant NSCLC cell lines and a pilot study on the effects of a combined MET and EGFR inhibitors treatment. The characterization of the erlotinib-resistant cell lines confirmed the association of EGFR TKI resistance with loss of *EGFR* gene amplification and/or AXL overexpression and/or *MET* gene amplification and MET receptor activation. These cellular models can be instrumental to further investigate the signaling pathways associated to EGFR TKI-resistance. Finally the drugs combination pilot study shows that *MET* gene amplification and MET receptor activation are not sufficient to predict a positive response of NSCLC cells to a cocktail of MET and EGFR inhibitors and highlights the importance of identifying more reliable biomarkers to predict the efficacy of treatments in NSCLC patients resistant to EGFR TKI.

## Introduction

Epidermal growth factor receptor (EGFR), member of the human epidermal growth factor receptor (HER) family controls key cellular programs, including survival, proliferation, differentiation and migration during development and adult life [1, 2]. *EGFR* gene is either mutated or shows altered expression in a variety of human cancers. Lung is the most frequent cause of cancer-related mortality worldwide leading to over a million deaths each year [3]. Based on histological characteristics, the two principal types of human lung cancer are small cell lung cancer (SCLC) and non small cell lung cancer (NSCLC); the latter being the most commonly detected type contributing to nearly 85% of cases. Identification of all driver oncogene alterations in lung adenocarcinoma and consequently adoption of molecular target therapies is challenging because of a large burden of passenger events per tumor genome [4–7]. NSCLC patients, whose tumors harbor EGFR sensitizing mutations in exon 19/21, get a meaningful clinical benefit from EGFR TKI treatments. However, despite an initial response to these inhibitors, most patients ultimately develop drug resistance, followed by relapses [8–18]. Several clinical studies have shown that a secondary mutation in the tyrosine kinase domain of EGFR (T790M) is responsible for the development of resistance to EGFR-targeting TKIs in approximately half of the cases of lung adenocarcinoma [19–21]. Acquired NSCLC resistance to TKIs has also been associated to overexpression and activation of other receptor tyrosine kinases (RTKs) including HER3, AXL or MET [22–26], to modulation of Fas receptor and NF-kB signaling pathways [27] and to epithelial to mesenchymal transition (EMT) [28–30].

The MET receptor and its ligand, hepatocyte growth factor (HGF), have recently been identified as novel promising targets in several human malignancies, including NSCLC. MET receptor mediates multiple biological responses promoting tissue remodeling, wound repair, organ homeostasis and cancer metastasis. In several solid tumors, *MET* gene amplification, mutations or overexpression lead to constitutively activated MET receptor [31, 32]. *MET* amplification occurs in 5–20% of NSCLC patients and its amplification or up-regulation correlates with acquired resistance to EGFR TKI treatments [25, 26, 33]. MET amplification can occur in NSCLC also before treatment with TKIs [34]. For all above reasons MET could become a valuable target for cancer therapy and several drugs targeting MET or its ligand HGF are currently undergoing early phase clinical trials in various cancers [35–37].

The identification of model systems to investigate or validate strategies to disrupt EGFR-dependent tumor cell growth is critical and may provide the basis for clinical applications. Therefore we decided, as others, to develop and characterize NSCLC cell lines resistant to EGFR TKIs. In our cellular model system we observed, as previously reported, association of EGFR TKI-resistance with loss of EGFR mutated allele amplification and/or AXL overexpression and/or *MET* gene amplification and MET receptor activation. Moreover, we demonstrated that *MET* gene amplification and MET receptor activation are not sufficient to predict a positive effect of a combination of MET and EGFR inhibitors in erlotinib resistant NSCLC, suggesting the need of identifying other biomarkers in order to move towards a precision medicine treatment in NSCLC patients.

## Material and Methods

### Material

#### Cells, Antibodies, and Reagents

The human cell lines: HCC827 (ATCC® CRL-2868™) and HCC4006 (ATCC® CRL-2871™), kindly provided by Oreste Segatto, were cultured in RPMI 1640 medium (BioWhittaker, Lonza, USA) supplemented with 10 mM Hepes pH 6.98–7.30, 1 mM L-glutamine, 100 U/ml penicillin/streptomycin (BioWittaker, Lonza) and heat inactivated 10% fetal bovine serum (FBS) (Sigma-Aldrich). All cells were cultured at 37°C in a 5% CO_2_ humidified incubator. Erlotinib (ERL)-resistant cell lines (RA1, RA2, RB1, RB1.1, RB2 and RC2.2) established essentially as previously described [29] were cultured in the same experimental conditions. Briefly, HCC827 and HCC4006 parental cell lines were cultured in complete tissue culture medium with a stepwise increase of erlotinib concentrations (stepwise method), or a high concentration of erlotinib (1 μM) (high-concentration method) over 5–6 months.

The primary antibodies: EGFR (clone D09, kindly provided by O. Segatto); phosphorylated-EGFR (Tyr1068); HER2/ErbB2 (D8F12); HER3/ErbB3 (D22C5); HER4/ErbB4 (111B2); phospho-HER2/ErbB2 (Y1221/1222) (6B12); phospho-HER3/ErbB3 (Y1289) (D1B5); phospho-HER4/ErbB4 (Y1284) (21A9); p44-42 MAPK (ERK1/2) (#9102), phospho-p44-42 MAPK (ERK1/2) (T202/Y204) clone E10; c-MET (D1C2); phospho-c-MET (Y1234/1235) (D26); Akt and phospho-Akt (S473), clone D9E were from Cell Signaling Technology (CST); AXL (#AF154) and phospho-AXL (Y779) (#AF228) were from R&D Systems; GAPDH, clone 1D4 or #5174 was respectively from Novus Biologicals or CST. Secondary antibodies: goat anti-rabbit IgG (H+L)-HRP and goat anti-mouse IgG (H+L)-HRP were from Bio-Rad; donkey anti-goat IgG-HRP (sc 2020) was from Santa Cruz Biotechology; streptavidin Alexa Fluor-488 were from Life Technologies. Biotin-labeled horse anti-rabbit Ig was from Vector Laboratories Burlingame, CA, USA. Tyrosine kinase inhibitors: Erlotinib Hydrochloride Salt, gefitinib Free Base, Vandetanib Free Base Lapatinib, Imatinib and Paclitaxel were from LC Laboratories, USA; SU11274, PHA-665752 hydrate and PF-04217903 were from Sigma-Aldrich, AZD9291, Rociletinib (CO-1686, AVL-301) and R428 (BGB324) were from Selleckchem and distributed by DBA Italia. Stock solutions of 10 mM for all inhibitors were prepared in DMSO and stored at -20°C or -80°C. MTT, 3-(4,5-methylthiazol-2-yl)-2,5-diphenyltetrazolium bromide was from Sigma-Aldrich. MTT stock solution (5 mg/ml in H_2_O, sterilized by filtration) was stored at 4°C for 1 months. Power SYBR Green PCR Master Mix was from Applied Biosystems. TRIzol reagent was from Life Technologies, Reverse Transcription System was from Promega.

### Methods

#### Cell growth inhibition MTT assay

Cells (10–20.000 cells/well) plated in 96-well plates at day 0 were treated at day 1 with increasing concentrations of TKIs (from 64 pM up to 10–20 μM if not otherwise stated) in complete tissue culture medium and cultured for 72 hours at 37°C in 5% CO_2_. Next, cells were gently washed with 1x PBS, incubated for 4 hours with MTT and processed for color detection with DMSO. The resulting purple solution was spectrophotometrically measured at 570 nm as previously described [38–39]. The optical density values, obtained by MTT assay reading, of cells treated with drugs were expressed as percentage of cell survival and normalized with the value of cells treated with vehicle (DMSO). For the EGFR stimulation, serum starved cells (24 hours) were treated, in RPMI supplemented medium, with EGF (100 ng/ml) or vehicle (DMSO) for 8 minutes or 100 nM erlotinib for 30 minutes, 1–3 hours. Next, cells were harvested in 1x PBS supplemented with 0.5 mM Na_3_VO_4_.

#### Western blot analysis

Total cell lysates were prepared by rinsing the cells 2 times in ice cold PBS 1x and by using ice-cold RIPA buffer (50 mM Tris-HCl pH 7.5, 150 mM NaCl, 1% NP40, 0.25% sodium deoxycholate, 1 mM EDTA) containing a combination of protease and phosphatase inhibitors (1 μg/ml aprotinin, 2 μg/ml leupeptin, 10 mM NaF, 20 mM β-glicerophosfate, 10 mM Na pyrophosphate, 1 mM PMSF, 1 mM Na_3_VO_4_). EGFR stimulated cells were lysed in 50 mM Hepes pH 7.5, 150 mM NaCl, 5 mM EGTA, 1% Triton X100 and proteases and phosphatase inhibitors. Cell lysates were quantified for proteins content with the Bio-Rad DC Protein Assay kit.

Cell lysates (25–40 μg) were separated by 8–10% SDS-PAGE and transferred to nitrocellulose membranes 0.45 μm (GE Healthcare Life Sciences). The membranes were blocked with 5% non-fat milk in 1x TBS pH 7.6–8.0 containing 0.1 or 0.2% Tween 20 (TBST) or 2% BSA (Sigma-Aldrich) for 2 hour at room temperature (RT) and subsequently probed with primary antibodies in 5% non-fat milk or 2–5% BSA in TBST, as recommended by the manufacturer, overnight at 4°C. Then membranes were washed 10 minutes for 3 times with TBST and probed with horseradish peroxidase-conjugated secondary antibodies in 5% non-fat milk in TBST for 1 hour at RT. Chemidoc XRS Bio-Rad was used for images acquisition with a chemi-luminescent camera, band signals were quantified using ImageLab 4.0 Bio-Rad software.

#### Soft agar assays

Soft agar assays were performed essentially as previously described [40]. Briefly 1.5 ml of 0.7% of Bacto-Agar (Becton Dickinson) in RPMI medium was plated in 35 mm petri dishes (bottom layer). Next, cells (20.000 cells/plate), in 0.35% Bacto-Agar, were plated on each bottom layer (top layer). Cells were cultured at 37°C in a 5% CO_2_ humidified incubator for 2–3 weeks. Plates were stained with a 0.005% crystal violet/20% methanol solution and images recorded with an Olympus XM10 camera and processed using Olympus CellSens Standard 1.8.1 software. Finally colonies with a diameter >50 μm were counted with the ImageJ software. The percentage of colony forming efficiency (CFE) was calculated according to the formula: (number of colonies formed/number of cells seeded) x 100. Approximately 500–1000 colonies/plates; 4/7 plates for each cell lines were recorded and analysed by Image J software.

#### Ethics statement

All animal studies were carried out in accordance to experimental protocols as reviewed and approved by the CNR-IBCN animal care and use committee and the Public Veterinary Health Department of the Italian Ministry of Health (Rome, Italy) (IBCN-CNR– 0003357) according to the ethical and safety rules and guidelines for the use of animals in biomedical research provided by the relevant Italian laws and European Union’s directives.

#### Xenograft in nude mice

Athymic nude female mice (Foxn1^nu^/Foxn1^+^) (Harlan Laboratories) were housed in individually ventilated cages (IVC) under controlled conditions (20–22°C; 55–65% relative humidity; 12/12 hours light/dark cycle; irradiated standard diet and water *ad libitum*). To generate tumor xenografts, groups of 3–5 mice 5–10 weeks old were injected subcutaneously with NSCLC tumor cells (5–15 x 10^6^ cells in 200 μl 1x PBS) into the dorsal flanks of each mouse. Two independent experiments were performed with each NSCLC cell line. Tumor volume was calculated by caliper measurements of tumor length (L) and width (W) according to the formula: LxW^2^/2. Tumor size and body weight were measured twice per week. Differences in tumor sizes formed on both flanks of mice injected with erlotinib resistant cell lines were compared to their parental counterpart. To evaluate time to fold tumor volume increase, normalization of tumor volumes was done to the average tumor volume at day 3, within the experiment and across xenograft groups. Times to four and six fold increase from initial tumor volumes (day 3) were assessed. When the tumor volumes reached an average of approximately 0.6–0.8 cm^3^ mice, previously euthanized with intra-peritoneal injections of Tiletamine/Zolazepam (800 mg/kg) and Xylazine (100 mg/kg), were sacrificed and tumors were harvested, measured, photographed, and pathologically examined.

Excised tumors were fixed with 3.7% paraformaldehyde (PFA) (wt/vol) 1x PBS for 20 min at room temperature and then embedded in paraffin. Serial sections 8 μm thick were cut from the paraffin embedded tissue blocks and floated onto charged glass slides (Super-Frost Plus, Fisher Scientific, Pittsburgh, PA) and dried overnight at 60°C. Sections were deparaffinized in changes of xylene and rehydrated in decreasing concentrations of ethanol and rinsed in 1× PBS. For antigen retrieval, samples were boiled for 10 min in 10 mM sodium citrate buffer (pH 6.0) and cooled for 5–10 min in water. Slides were washed in 1× PBS and incubated with blocking buffer (1× PBS, 0.1% Triton X-100, 1% BSA, 4% donkey serum) for 1 h and then incubated overnight at 4°C with primary antibodies diluted in blocking buffer. The following day slides were washed three times with washing buffer (1× PBS, 0.1% Triton X-100), incubated with biotinylated anti-Ig secondary antibody for 5 h followed by streptavidin Alexa 488, and finally washed as before and mounted using Mowiol 4–88 mounting media. Nuclei were stained using Hoechst 33258 (Sigma Aldrich). For xenograft studies, all tumors were also stained with the omission of primary antibody as a negative control. For confocal analysis Argon ion laser at 488 nm and blue diode laser at 405 were used as excitation sources. Confocal Z-stacks were collected at 0.5 μm intervals to a total optical depth of 8–10 xm. Confocal images were processed with Volocity (Improvision, Perkin Elmer) and Adobe Photoshop CS4 software for image rendering and representation of x/y view. Images for direct comparison were collected under same parameters and representative images were chosen.

#### Genomic DNA preparation

Cell pellets (approximately 10x10^6^ cells) were lysed in 10 mM Tris-HCl pH 8.0, 200 mM NaCl, 2 mM EDTA pH 8.0. Then lysates were brought to 400 mM NaCl, 0.6% SDS, 300 μg/ml proteinase K and incubated overnight at 55°C in water bath. Saturated NaCl (0.3 volumes) was added and the samples were vigorously mixed followed by spinning at 14000 rpm at 4°C for 15 minutes. The supernatant was precipitated and the DNA dissolved in TE buffer (10 mM Tris-HCl pH 7.5, 1 mM EDTA). Afterwards genomic DNA was treated with RNAse A to remove contaminant RNA. Genomic DNA was incubated in 300 μl of TE buffer and 15 μl of RNAse A (1 μg/μl) in a 65°C water bath for 10 minutes. Then, RNAse A was precipitated with 0.4 volumes of 7.5 M ammonium acetate for 30 minutes at 4°C, centrifuged at 13000 rpm for 30 minutes at 4°C. The supernatant was precipitated with absolute ethanol. After washes with 70% ethanol, genomic DNA was resuspended in TE buffer.

#### Mutation analysis by direct sequencing

EGFR exons 19 and 20 and KRAS exons 2 and 3 were amplified by PCR (for primer pairs see S1 Table). Amplified products were then purified using Exostar 1-Step (VWR International) according to the manufacturer's instructions. Sequencing reactions were performed using the Big Dye Terminator version 3.1 (Applied Biosystems, Foster City, CA, USA). Dye purification was carried out by Centrisep Spin columns (Princeton Separation) and subsequent sequencing analysis was resolved on a 3130XL Genetic Analyzer (Applied Biosystems). Sequences were finally analyzed with Sequence Analysis v5.2 and SeqScape v2.5 (Applied Biosystems).

#### Relative quantitation of gene copy number

Differences in EGFR and *MET* gene copy number between ERL-resistant and parental cell lines were determined by quantitative real-time PCR (qPCR) using an Applied Biosystems 7500 Fast Real-Time PCR System (Applied Biosystems). PCRs were carried out in 20 μl volume containing 5 ng of genomic DNA, 200 nM each primer for *MET*, *EGFR* and *Ribonuclease P* (*RNase P)*, in independent reactions (S1 Table) and 1x Power SYBR Green PCR Master Mix (Applied Biosystems). PCRs for each primer set were performed in triplicate, and mean values were calculated. Quantification was based on the standard curve method. *RNase P* was used as a reference gene, to normalize quantitation of target genes for differences in the amount of total DNA in each sample. Genomic DNA of HCC827 and HCC4006 parental cell line were used as calibrator samples, relative to which differences in gene copy number have been calculated. The data were analyzed using SDS (Ver. 1.4) software (Applied Biosystems).

#### RNA analysis

For quantitative RT-PCR analysis, total RNA was extracted using the TRIzol (Life Techologies) reagent and retro-transcribed with the Reverse Transcription System (Promega) using oligo (dT) and random primers. qPCR analysis was performed using a 7500 Fast Real-Time PCR System (Applied Biosystems). PCRs were carried out in 20 μl volume containing 10 ng of total RNA, 200 nM of each primer and 1X Power SYBR Green PCR Master Mix (Applied Biosystems). *Ribosomal protein L31* (*rp-L31*) was used as a reference gene, to normalize quantitation of target genes for differences in the amount of total RNA in each sample. Total RNA of HCC827 and HCC4006 parental cell lines were used as calibrator samples, relative to which differences in the RNA amount of resistant cell lines have been calculated. The data were analyzed using SDS (Ver. 1.4) software (Applied Biosystems).

#### Drug combination studies and synergy quantification

The synergy of erlotinib and MET or erlotinib and AXL inhibitors was evaluated by the Chou-Talalay method [41, http://www.combosyn.com]. In brief, the cells were treated with 8–12 concentrations of erlotinib each in combination with 8–12 concentrations of MET or AXL inhibitors. In particular the ratio of erlotinib with SU11274 was 1:4, while the ratio of erlotinib with PHA-665752 or PF-04217903 was 1:1. The ratio of erlotinib with the AXL inhibitor was 10:1 as previously reported [42]. Erlotinib was used at a concentration approximately equal to its IC_50_ in HCC827 cell line and at concentrations within 5-fold increments above or below. Each drug was also used alone at the same concentrations. Cell survival was determined by MTT assays. Each data point was performed in triplicates. The CompuSyn software (ComboSyn Inc., Paramus, NJ) was used to determine dose-effect curves for single and combination treatments. Further, combination index (CI) values were calculated to assess the nature of drug interactions that can be, additive (CI = 1), antagonistic (CI>1) or synergistic (CI<1). In particular, nonlinear regression trendlines were used to calculate CI. Single and combination dose response curves were entered into CompuSyn software and the following equation was used: [(D1/Dx1)+(D2/Dx2)], in which Dx1 is the dose of Drug 1 that inhibits cell survival at x% and Dx2 is the dose of Drug 2 that inhibits cell survival at x% and D1 is the portion of Drug 1 that also inhibits cell survival at x% in combination with Drug 2 and vice versa. The doses which produced a particular effect (Dx) can be calculated from the Median effect equation: Dx = Dm [fa/1-fa)]1/m, where, *Dm*, *f*_a_ and *f*_u_ (1-fa) represent: the median dose, fraction affected and fraction unaffected, respectively. *Dm* was estimated from the antilog of the *X*-intercept of the median effect plot, where *X* = log (*D*) versus *Y* = log (*f*_a_/*f*_u_); which means *Dm* = 10^−(Y-intercept)/m^, *m* being the slope of the median effect plot.

#### Statistical analysis

GraphPad Prism software 6.0c was used for MTT data analysis. The regression trendline were fitted using a non-linear regression method and IC_50_ values were determined using a sigmoidal dose response inhibition variable slope method.

In the drug combination studies, dose–effect curve parameters, CI values, Fa-CI plot (plot representing CI versus Fa, the fraction affected by a particular dose) were calculated by CompuSyn program (Compusyn Inc, Paramus, NJ, USA).

In the xenograft experiments the statistical significance of the results was evaluated by two way analysis of variance and Bonferroni multiple testing to compare differences in tumor fold growth between ERL-resistant and HCC827 parental cell line. All statistical tests were performed using GraphPad Prism and the threshold for statistical significance was set at P-values lower than 0.05.

## Results and Discussion

### Establishment of erlotinib resistant NSCLC cell lines

**Fig 1:**
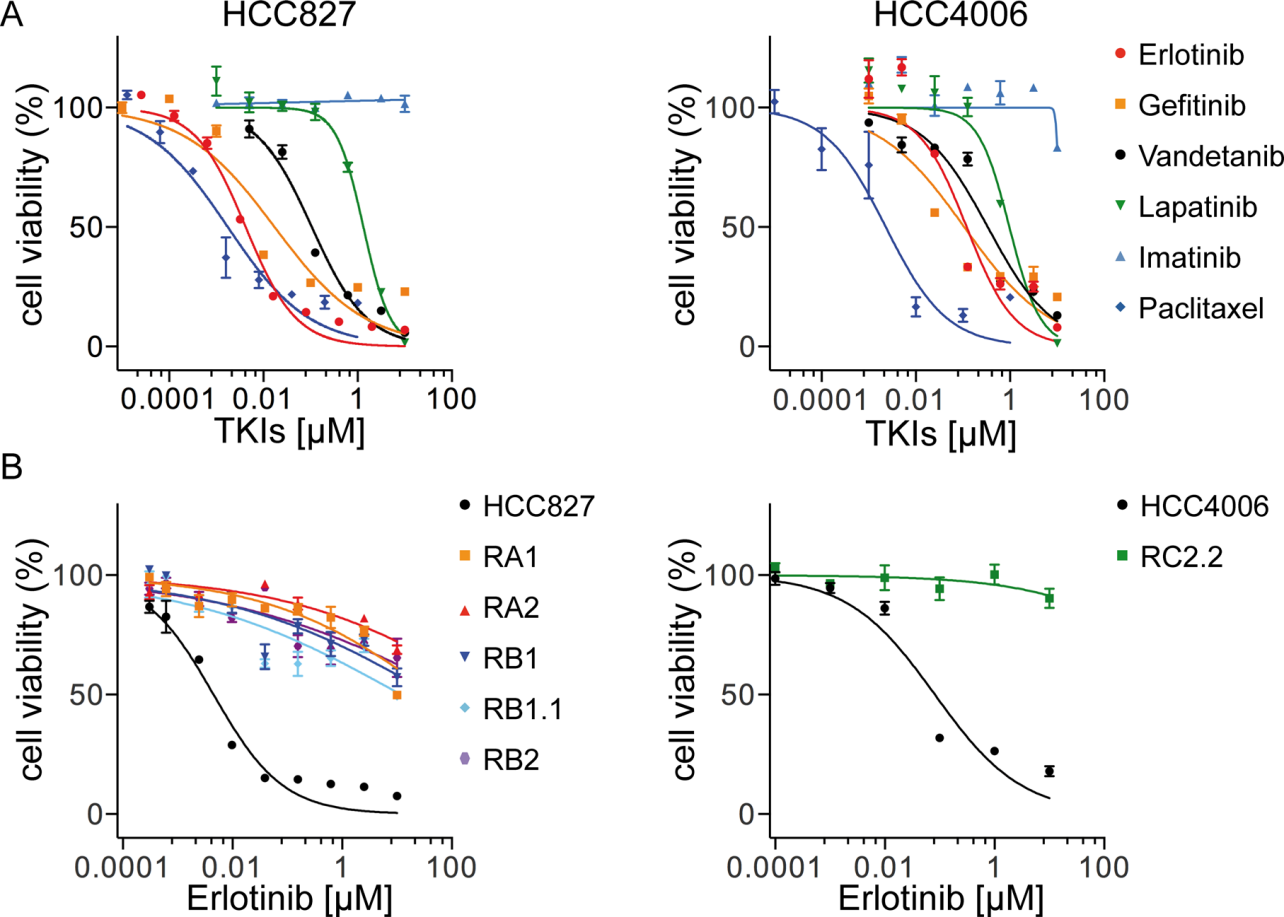
Cell inhibition growth analysis of ERL-resistant NSCLC cell lines. A) Representative dose-effect curve plots of HCC827 and HCC4006 parental cell lines to the indicated TKIs. Cell viability was determined by MTT assays. The results are expressed as the percentage of cell viability in drug-treated cultures relative to DMSO-treated control samples; B) Dose effect curve plots of derived ERL-resistant HCC827 and HCC4006 cell lines. The results are expressed as described above. Data (mean ± s.e.m) in A and B are representative of more than three independent experiments.

In order to investigate mechanisms leading to resistance to EGFR-targeted therapy, two NSCLC cell lines HCC827 and HCC4006 were used to derive *in vitro* models of acquired resistance to the *EGFR* TKI, erlotinib. Both cell lines harbor EGFR activating mutations in the tyrosine kinase domain, the HCC827 cell line a deletion in exon 19 (ΔE746-A750) and HCC4006 a deletion (ΔL747-E749) and a point mutation (A750P) in exon 19. HCC827 and HCC4006 cell lines are both highly sensitive to TKIs targeting the EGFR. TKI dose-response curves and IC_50_ values are shown respectively in Fig 1A, Table 1. Specifically, both cell lines respond to erlotinib [43] and gefitinib [44] with an IC_50_ in line with previous reports [26, 45]. Paclitaxel, a member of the taxane family and an important agent in cancer chemotherapy that acts by binding to microtubules and interfering with the mitotic process, was used as positive control [46]. Sensitivity to vandetanib [47–49] and to a lesser extent to lapatinib [50], TKIs targeting respectively VEGFR (Vascular Endothelial Growth Factor Receptor)/EGFR/RET and EGFR/HER2, was also observed. Imatinib, targeting mainly ABL, PDGFR (Platelet Derived Growth Factor Receptor) and c-KIT [51–53] had no effect on both HCC827 and HCC4006 cell lines (Fig 1 and Table 1).

**Table 1:** IC_50_ values of TKIs against parental and ERL-resistant NSCLC cell lines.

	HCC827	RA1	RA2	RB1	RB1.1	RB2	HCC4006	RC2.2
**Erlotinib**	4–17 nM	>10 μM	>10 μM	>10 μM	>10 μM	>10 μM	80–200 nM	>10 μM
**Gefitinib**	4–20 nM	>10 μM	>10 μM	>10 μM	>10 μM	>10 μM	25–107 nM	>10 μM
**AZD9291**	0.4–10 nM	>1 μM	>1 μM	>1 μM	>1 μM	>1 μM	1–8 nM	>1 μM
**Rociletinib**	12–32 nM	>1 μM	>1 μM	>1 μM	>1 μM	>1 μM	100–195 nM	>1 μM
**Vandetanib**	70–180 nM	>10 μM	>10 μM	>10 μM	>10 μM	>10 μM	310–390 nM	>10 μM
**Lapatinib**	0.5–1.7 μM	>10 μM	>10 μM	>10 μM	>10 μM	> 10 μM	0.65–1.85 μM	> 10 μM
**Imatinib**	>1 μM	-	-	-	-	-	>1 μM	-
**Paclitaxel**	0.1–2 nM	1–4 nM	>1 μM	1–4 nM	0.1–2 nM	1–5 nM	0.1–4 nM	> 1μM

It has been suggested that the features of drug-resistant cells may vary depending on the protocol used for the selection process [29]. Therefore we derived resistant cell lines by exposing HCC827 and HCC4006 cell lines (parental) to erlotinib for 5–6 months, following two distinct protocols essentially as previously described [29]. Briefly, in “protocol 1”, cells were exposed to increasing concentrations of erlotinib (2x IC_50_−25/50x IC_50_), while in “protocol 2” cells were continuously exposed to a high dose of erlotinib (1 μM). The selection outcomes were monitored periodically by cell growth inhibition assays by using cells in “drug holiday” for at least 1 week. Five erlotinib (ERL)-resistant cell lines were isolated from the parental HCC827 cell line, three with “protocol 1” (RA1, RB1, and RB1.1) and two with “protocol 2” (RA2 and RB2) and one cell line was obtained from HCC4006 cell line by using “protocol 2” (RC2.2). Several attempts to derived additional HCC4006 ERL-resistant cell lines with both protocols failed.

All cell lines derived from parental cell lines are resistant to erlotinib and gefitinib (IC_50_ > 10 μM) (Fig 1B, Table 1) as well to the third generation irreversible EGFR inhibitors AZD9291 [54] and Rociletinib (CO-1686, AVL-301) [55] now in advanced stage clinical trials (Table 1) and highly sensitive to paclitaxel with the exception of RA2 and RC2.2 cell lines that exhibited higher IC_50_ values for paclitaxel than those of parental cell lines (Fig 1B, Table 1). Interestingly, the derived ERL-resistant cell lines are also resistant to inhibitors targeting other RTKs besides EGFR (Table 1). Importantly the resistant phenotype is stable in the absence of drug selection pressure thus resembling the phenotype of cancer cells that could survive in patients in drug holiday.

**Fig 2:**
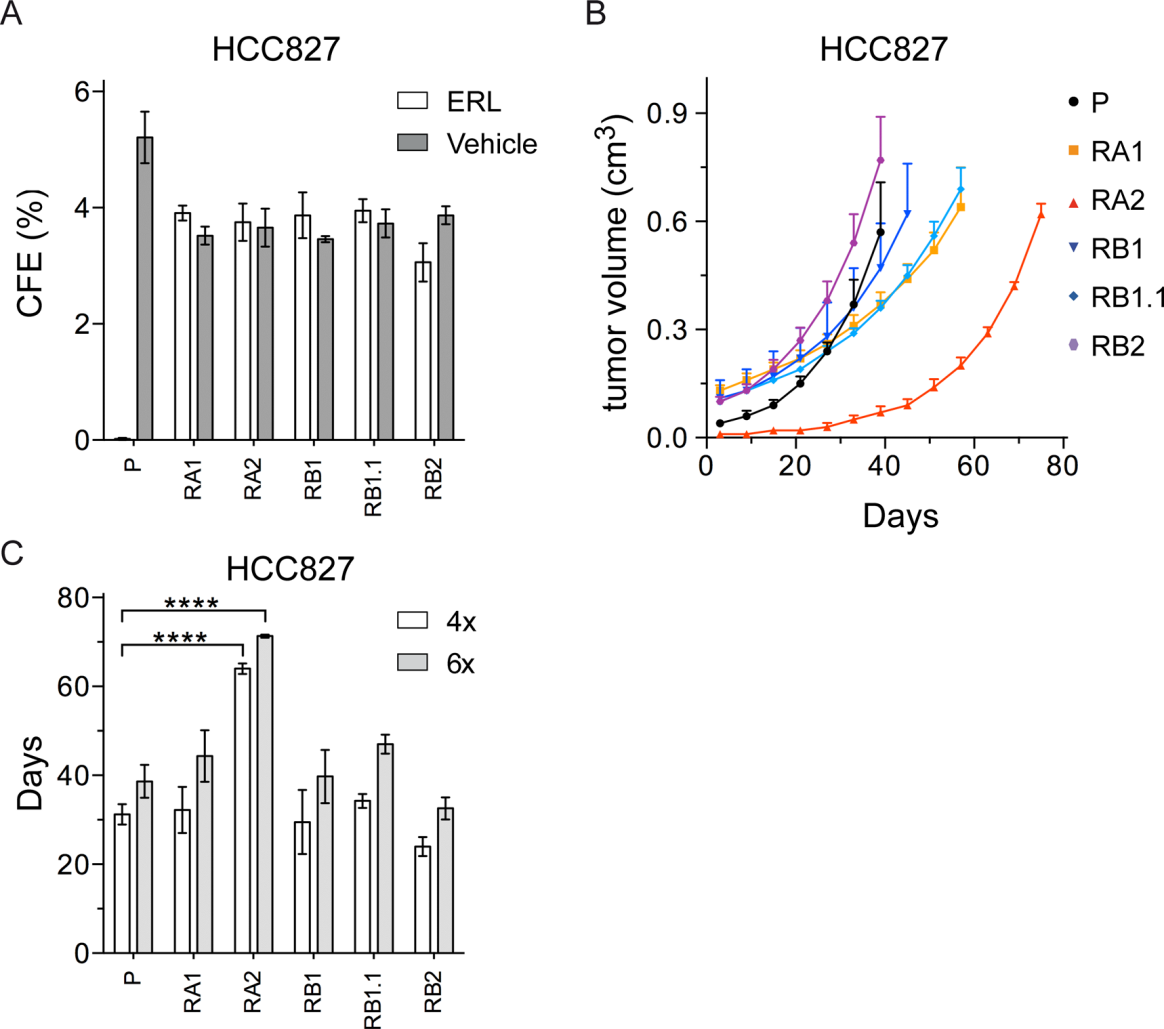
The ERL-resistant HCC827 cells lines are tumorigenic. A) Soft agar colonies were counted by ImageJ software. The percentage of colony forming efficiency (CFE) of parental (P) and ERL-resistant cell lines, as mean ± s.e.m., is shown. Histograms in gray and white colors indicate respectively vehicle (DMSO, 0.01%) and ERL (erlotinib 1 **μ**M) cell treatments; B) Tumor volume of xenograft nodes, calculated as described in material and methods, are shown as mean ± s.e.m; C) Times to four (4x) and six fold (6x) increases from initial tumor volumes (day 3) are shown respectively by white and grey bars. P-values < 0.0001 are indicated.

To investigate the tumorigenicity of the ERL-resistant cell lines and verify whether erlotinib could inhibit their anchorage-independent growth we performed soft agar colony formation assays. All HCC827 cell lines formed colonies similar in number and size (diameter > 50 μm), but only the ERL-resistant cell lines formed colonies in presence of erlotinib (Fig 2A). The HCC4006 and RC2.2 cells formed colonies but smaller in size (diameter < 40–50 μm) and with lower frequency. Nevertheless, the ERL-resistant RC2.2 cell line was resistant to erlotinib in soft agar as well (data not shown).

To further investigate the tumorigenicity of the NSCLC cell lines, we performed xenograft assays in athymic nude mice (Fig 2B). Similar tumor growth curves were recorded for parental and ERL-resistant HCC827 cell lines with the exception of RA2 xenografts that showed a relevant tumor growth delay (Fig 2B) with statistically different times required to reach a four and six fold increase from initial tumor volumes (Fig 2C). Since the RC2.2 cell line grows well in culture but at low cell density (1:5 when compared with the HCC827 parental and derived cell lines), xenograft assays were performed only with 5x10^6^ cells. In those experimental conditions, only small nodes and not in all mice were obtained (data not shown). Overall the data demonstrate that HCC827 ERL-resistant cell lines and, to a lesser extent, RC2.2 cell line are tumorigenic in anchorage-independent systems. Importantly, even if the selection protocols used monolayer tissue culture conditions, the derived NSCLC cell lines grow in presence of erlotinib in 3D model systems, more closely resembling *in vivo* tissues conditions.

### *EGFR* T790M or *KRAS* gene mutations are not present in the ERL-resistant NSCLC cell lines

**Fig 3:**
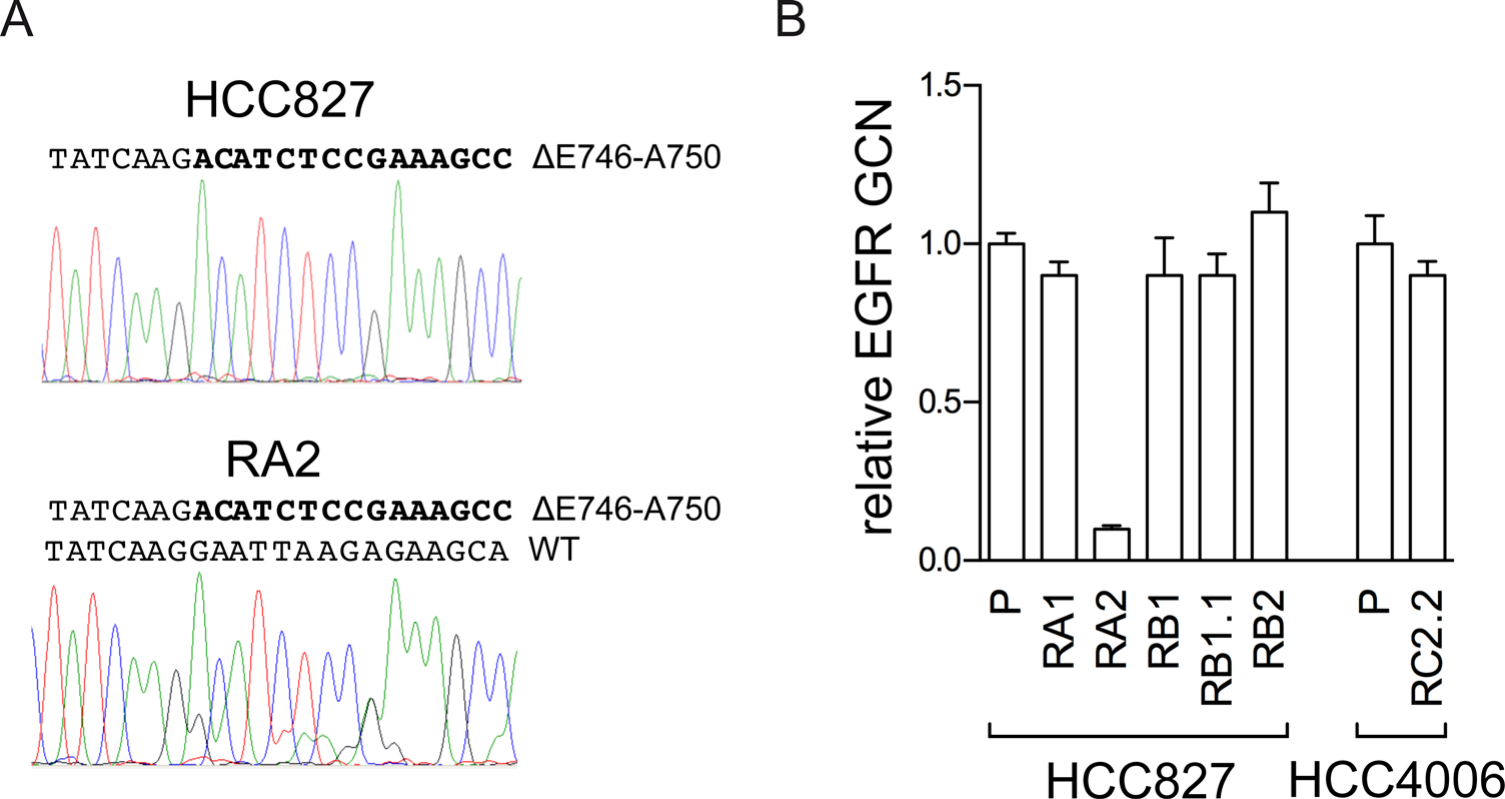
Analysis of the *EGFR* gene in the RA2 ERL-resistant cell line. A) Analysis of *EGFR* exon 19 nucleotides sequence. The pherogram of the parental cell line with peaks corresponding to the *EGFR* mutated sequence (ΔE746-A750) and the pherogram of the RA2 resistant cells with peaks corresponding to the mutated and wild type (WT) EGFR nucleotides sequence are shown. B) qPCR analysis. Relative *EGFR* gene copy number (GCN) in genomic DNA, normalized to the *Rnase P* gene, is expressed relative to the levels in parental cell lines (P) set as 1 (mean ± SD of triplicate determinations). Results are representative of those obtained from 2 independent analysis.

To verify *EGFR* and *KRAS* oncogene mutations, nucleotide sequence analysis of exons 19–20 of the *EGFR* gene and codons 12/13 and 61 of the *KRAS* gene was performed with specific primers listed in S1 Table. We confirmed the presence of mutations in *EGFR* exon 19 in both parental and ERL-resistant cell lines and we excluded the presence of an *EGFR* T790M gene mutation, the most common cause of acquired resistance to erlotinib in NSCLC patients (Table 2). Interestingly, the analysis of pherograms showed in HCC827 only the mutated allele, likely for its high amplification levels, while in RA2 both allele sequences were detected suggesting a loss of *EGFR* gene amplification in the mutated allele. Gene copy number analysis by qPCR confirmed a decrease in EGFR copy number in RA2 cell line (Fig 3). This cell line could be a useful tool to further investigate the dosage effect of *EGFR* mutated allele in erlotinib sensitivity. *KRAS* gene mutations appear to be mainly mutually exclusive with *EGFR* gene activating mutations [56–59], however co-occurrence of *KRAS* and *EGFR* activating mutations has been recently reported in Chinese and Indian NSCLC patients [60–61]. *KRAS* gene codons (12/13, 61) were wild type in all our NSCLC cell lines (Table 2).

**Table 2:** Analysis of *EGFR* and *KRAS* gene mutations.

	*EGFR* Exon 19	*EGFR* Exon 20	*KRAS* Exon 2 codons 12/13	*KRAS* Exon 3 codon 61
**HCC827**	ΔE746-A750	WT	WT	WT
**RA1**	ΔE746-A750	WT	WT	WT
**RA2**	WT/ΔE746-A750	WT	WT	WT
**RB1**	ΔE746-A750	WT	WT	WT
**RB1.1**	ΔE746-A750	WT	WT	WT
**RB2**	ΔE746-A750	WT	WT	WT
**HCC4006**	ΔE746-A750	WT	WT	WT
**RC2.2**	ΔE746-A750	WT	WT	WT

Cells were analyzed for the mutational status by nucleotides direct sequencing. Δ: E746-A750 deletion of exon 19, WT: wild-type.

### Erlotinib binds EGFR and impairs EGFR and ERK1/2 phosporylation

**Fig 4:**
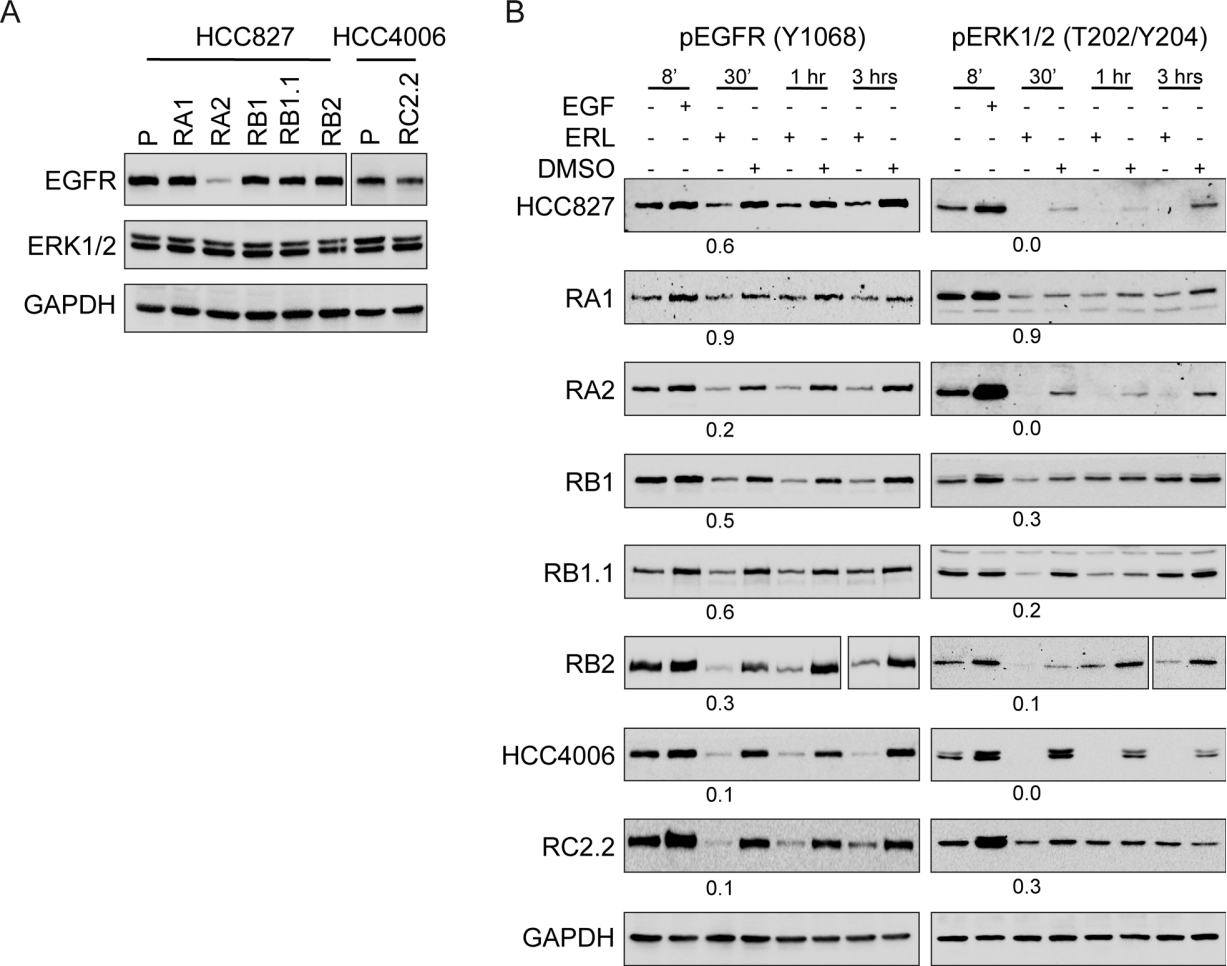
Erlotinib impairs EGFR and ERK1/2 phosphorylation in ERL-resistant cell lines. A) Representative western blots with EGFR and ERK1/2 antibodies and B) pEGFR (Y1068) and pERK1/2 (T202/Y204) antibodies in the indicated parental and ERL-resistant cell lines treated with EGF (100 μg/ml), ERL (Erlotinib, 100 nM) or vehicle (DMSO) at different time points (8’, 30’, 1hr, 3hrs). Densitometric analyses of band signals were normalized with GAPDH, the number indicates the signals quantification at 30’ upon ERL-treatment. For RA2 cell line double amount of total cell lysate was loaded to analyze EGFR expression.

By western blotting analysis we demonstrated that all ERL-resistant cell lines express similar levels of total and constitutively phosphorylated EGFR and ERK1/2, with the exception of RA2 cell line that showed a strong decrease in EGFR protein levels (Fig 4A). Upon erlotinib treatment a decrease in EGFR and ERK1/2 phosphorylation signals was observed (Fig 4B) indicating that erlotinib can still bind EGFR and that, unlikely, rare secondary mutations in the EGFR tyrosine kinase domain are present and/or can impair TKI binding. Furthermore, the mitogen activated protein kinase (MAPK) downstream targets of the EGFR signaling pathways, ERK1/2, are still responsive to erlotinib (Fig 4B). Therefore, while with a partial dephosphorylation of EGFR and ERK1/2 proteins we cannot exclude a contribution of EGFR activation due to intratumoral drug-response heterogeneity, bypass tracks signaling are likely involved in the ERL-resistant phenotype.

### HER2 and HER3 receptors are not overexpressed in ERL-resistant NSCLC cell lines

**Fig 5:**
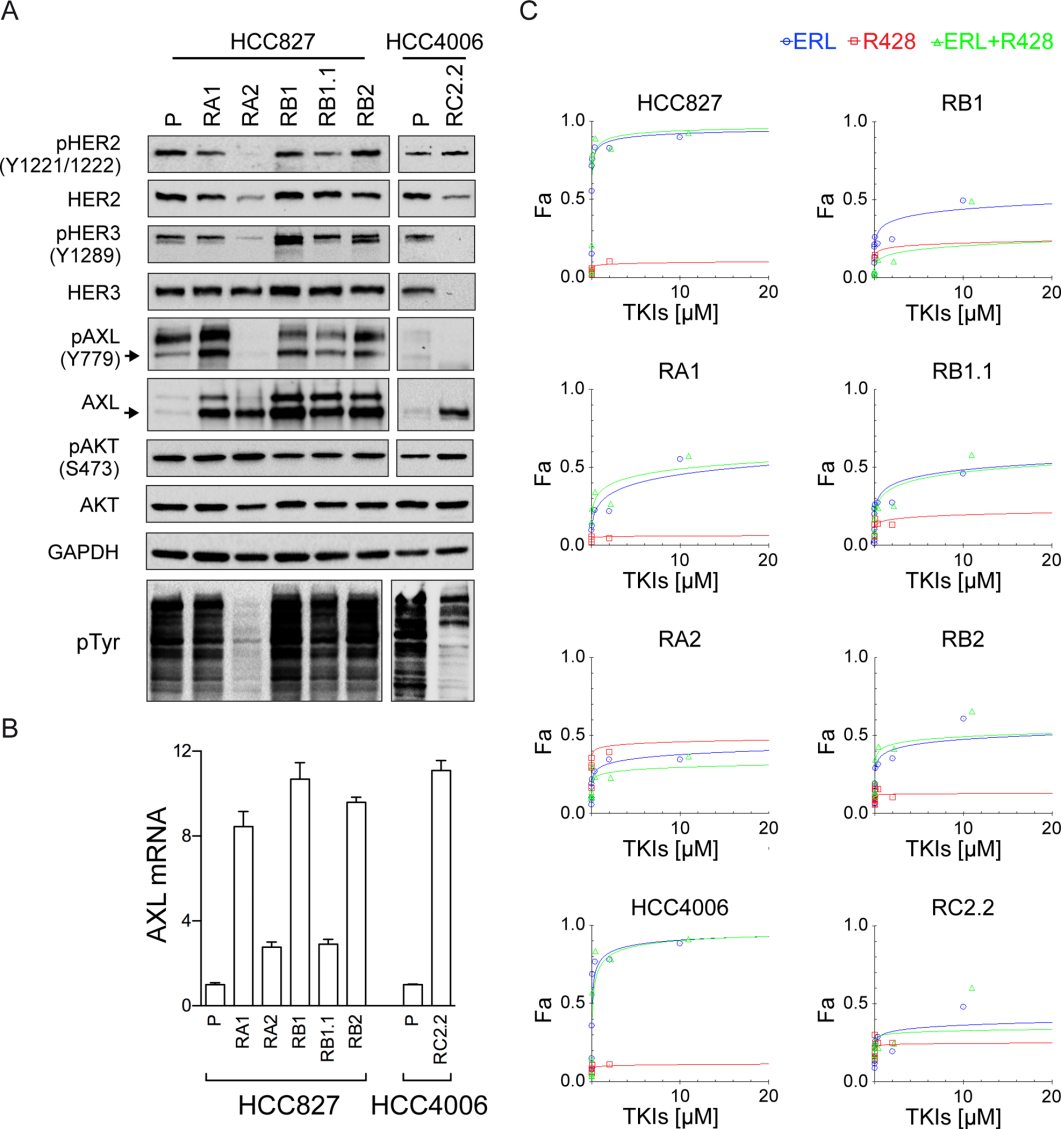
HER2/HER3 and AXL expression and phosphorylation analysis. A) Representative western blots of total cell lysates of HCC827 and HCC4006 parental cell lines (P) and their derived ERL-resistant cell lines. Arrows indicate the expected molecular weight size. Total cell lysates loaded were 40 μg for AXL and pAXL analyses and 25 μg for the others. B) qPCR analysis of AXL mRNA normalized to rp-L31 mRNA and expressed relative to the levels in parental cell lines set as 1 (mean ± SD of triplicate determinations). Western blots and qPCR data are representative of those obtained respectively from 3 and 2 independent analysis. C) Dose-effect curves were calculated using CompuSyn software and plotting the entered Fa values against the entered dose values. For combination treatments, the combined drugs dose was entered. Each data point represents the mean of 3 replicates.

Ligand binding to human HERs results in the formation of homo- or heterodimers that activate RTKs and subsequently downstream signaling pathways including the phosphoinositide 3-kinase (PI3K)/protein kinase B (AKT) [62]. HER2 amplification and elevated expression of HER3 are frequently observed in various malignancies including NSCLC [63, 64]. HER3 can promote tumor progression via interactions with other RTKs due to its lack of or weak intrinsic kinase activity. To investigate the expression and phosphorylation status of all HER family members in our model system we performed western blot analysis using parental and ERL-resistant cell lines. HER2 and HER3 signals were similar in parental and ERL-resistant cell lines with the exception of RA2 and RC2.2. In particular, RA2 showed very low HER2 expression and RC2.2 low HER2 and undetectable HER3 signals (Fig 5A). Constitutive phosphorylation of HER2 (Y1221/1222) and HER3 (Y1289) was detected in all cell lines with the exception of RA2 and RC2.2 cell lines that showed undetectable or very low signals for pHER2 and pHER3 (Fig 5A). Notably, the total level of tyrosine phosphorylation was strongly and reproducible reduced in both RA2 and RC2.2 cell lines implying a marked deregulation of kinases and/or phosphatases in both cell lines (Fig 5A). HER4 protein was not expressed in parental and ERL-resistant cell lines (data not shown). Overall, these data suggest that HER2 and HER3 are not overexpressed and therefore unlikely responsible for the ERL-acquired resistance.

### AXL and MET receptor in ERL-resistant NSCLC

Among RTK signaling pathways, previously reported to be involved in mechanisms of ERL-resistance, we focused our studies on AXL and MET receptors.

AXL is considered a potential relevant target in NSCLC therapy and targeting strategies with TKIs, aptamers or molecules modulating its turnover are under very active investigation [65–68]. Moreover, an association of AXL deregulation to ERL-resistance in NSCLC has been previously reported [24].

AXL expression analysis by western blotting (Fig 5A) and qPCR (Fig 5B), in our model system, showed an overexpression of AXL in both HCC827 and HCC4006 ERL-resistant cell lines independently from the protocol used for selection. However, AXL was constitutively phosphorylated (Y779) in both parental and ERL-resistant HCC827 cell lines with the exception of RA2 (Fig 5A) and not phosphorylated in the RC2.2 cell line. To further investigate the possible role of AXL on ERL-resistance, cell viability inhibition studies were performed with a selective AXL inhibitor, R428 [42, 69]. TKI dose-response curves and Dm_50_ values indicate that AXL inhibitor alone or in combination with erlotinib did not impair the cell viability of ERL-sensitive and -resistant cell lines (Fig 5C, Table 3). Overall the data suggest that unlikely, in our cell lines, AXL activation is a key player in the mechanism of ERL-resistance.

**Table 3:** Dm_50_ of single agent and drugs combination for parental and ERL-resistant NSCLC cell lines.

	HCC827	RA1	RA2	RB1	RB1.1	RB2	HCC4006	RC2.2
**ERL**	6–20 nM	>10 μM	>10 μM	>10 μM	>10 μM	>10 μM	55–61 nM	>10 μM
**R428**	>1 μM	>1 μM	>1 μM	>1 μM	>1 μM	>1 μM	>1 μM	>1 μM
**ERL+R428**	7–11 nM	>10 μM	>10 μM	>10 μM	>10 μM	>10 μM	47–127 nM	>10 μM

Median effect concentrations (Dm_50_) values are indicated. ERL: Erlotinib

We also analyzed the expression levels and constitutive phosphorylation of the serine-threonine kinase AKT. AKT protein was expressed at similar levels in all parental and ERL-resistant cell lines. Similar constitutive phosphorylation levels of pAKT was observed in all HCC827 ERL-resistant cell lines, a slight and reproducible higher pAKT signal was observed in RC2.2 cell lysates when compared with the HCC4006 cell lysates by western blotting (Fig 5A).

**Fig 6:**
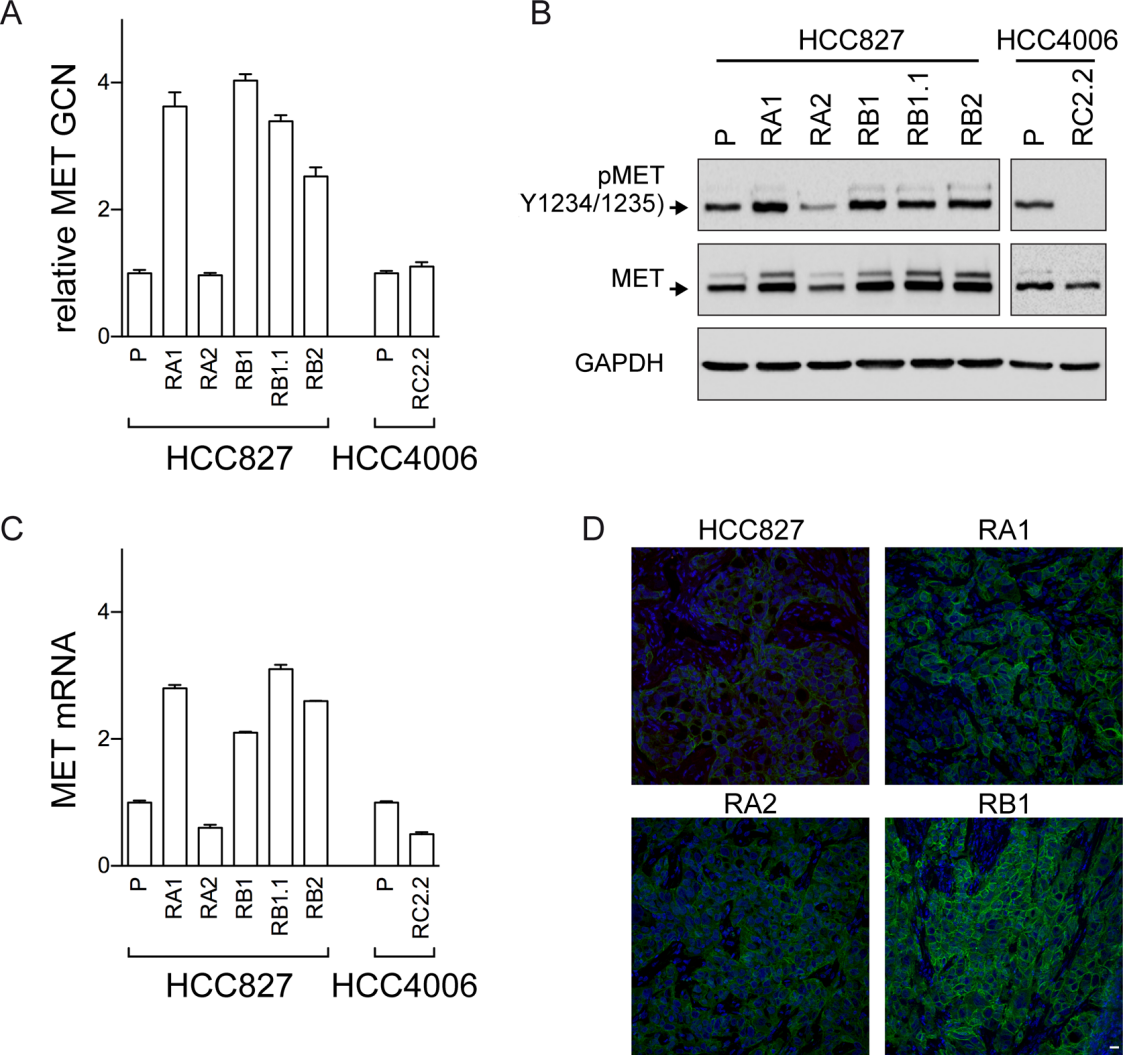
MET analysis in ERL-resistant cell lines. A) qPCR analysis of gene copy numbers of *MET;* B) western blots of total cell lysates with the antibodies indicated of parental (P) and ERL-resistant cell lines; C) qPCR analysis of MET mRNA expression in parental (P) and ERL-resistant cell lines. *MET* gene and mRNA in A) and C) are normalized to RNaseP gene and rp-L31 mRNA respectively and both are expressed relative to the levels in parental cell lines set as 1 (mean ± SD of triplicate determinations). qPCR data are representative of those obtained from 2 independent analysis; D) Confocal microscopy analysis of MET receptor (green) expression in xenograft nodes of mice subcutaneously injected with the parental HCC827 cells and the ERL-resistant RA1, RB1 and RA2 cell lines. Images show representative xy-plane maximum projection of the specimens. Scale bars correspond to 15 μm.

MET has recently emerged as a promising target in NSCLC and targeting strategies are actively explored in pre-clinical models and in ongoing clinical trials [35–37]. In our cellular model system we detected an increase in *MET* gene copy numbers by qPCR in all ERL-resistant cell lines with the exception of RA2 and RC2.2 cell lines (Fig 6A). MET protein and mRNA expression, respectively by western blot (Fig 6B) and qPCR (Fig 6C) analysis, confirmed the higher expression levels of MET in all cell lines harboring *MET* gene amplification. MET receptor was also constitutively phosphorylated at the Y1234/1235 site in all ERL-resistant cell lines with the exception of RC2.2 cell line (Fig 6B). Finally, immunohistochemistry studies of xenograft node sections, obtained by mice injected with HCC827 parental and ERL-resistant cell lines, confirmed the increased expression of MET in the ERL-resistant cell lines harboring amplified *MET* gene such as RA1 and RB1 when compared with parental and RA2 cell lines xenograft nodes (Fig 6D). Overall these data confirmed the association of ERL-resistance with *MET* gene amplification and overexpression as previously reported [26, 33].

### MET overexpression and activation are not sufficient to predict efficacy of EGFR and MET inhibitors

**Fig 7:**
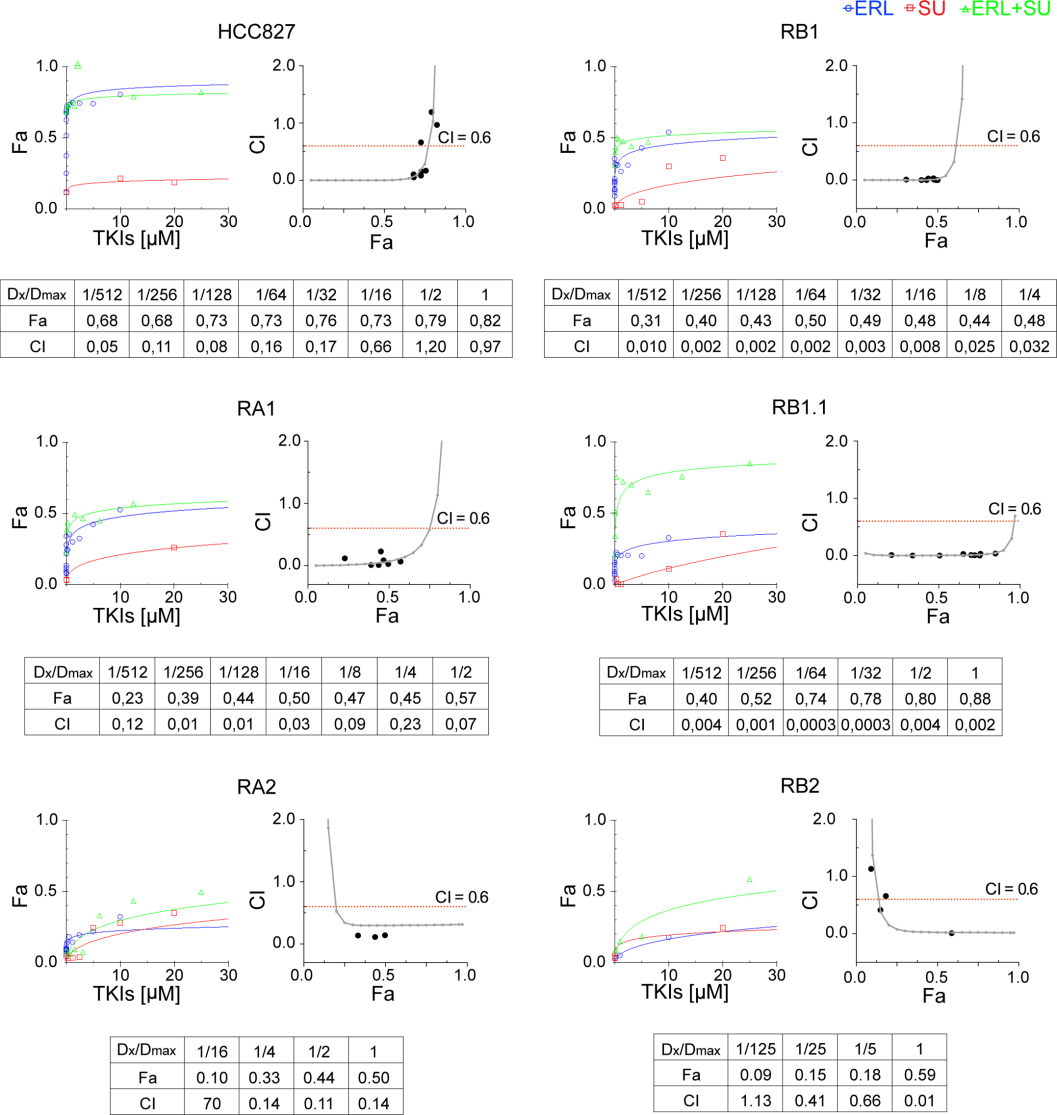
Synergistic effects of Erlotinib and MET inhibitors in ERL-resistant NSCLC cell lines. Dose-effect curves were calculated using CompuSyn plotting the entered Fa values against the entered dose values. For combination treatments, the combined drugs dose were entered. Each data point represents the mean of 3 replicates. Combination index (CI) values were generated by non-linear regression method. Trendlines indicate CI values at any given effect (Fa, fraction affected, %inhibition), actual data points are also shown. CI = 1, additivity; CI >1, antagonism; CI<1, synergy.

Several of our HCC827 ERL-resistant cell lines showed *MET* gene amplification and constitutive MET receptor activation, therefore we decided to investigate the effect on cell growth of the following specific MET inhibitors: SU11274 (SU) [70–72], PHA-665752 (PHA) [73–74], and PF-04217903 (PF) [75–77], and to determine their type of interaction with EGFR inhibitor. Briefly, sensitive and ERL-resistant HCC827 cell lines were treated with SU or erlotinib singly or in combination using 5-fold dilution series. For combination treatments, a fixed concentration ratio 1:4 of erlotinib and SU was used. Cell viability data were determined by MTT and entered as fractional effect (Fa) values into CompuSyn software. Fa values were plotted against the concentration of single drug or drug combinations; representative dose effect curves are shown in Fig 7. The data indicate that treatment with either MET inhibitor or erlotinib did not impair the viability of HCC827 ERL-resistant cell lines while treatments with both TKIs strongly inhibited the RB1.1 cell line growth. Importantly the RB1.1 showed very low Dm_50_ similar to the one recorded in the ERL-sensitive parental cell line (Table 4). Further calculated CI values between 0 and 2 were plotted against Fa values; representative Fa-CI plots are shown in Fig 7. Fa values as well as CI values for the actual experimental data points are shown along with the drug concentrations used for each point as ratio of the actual dose (Dx) and the maximal dose (Dmax) used (Fig 7). CI analysis indicated that while the drugs combination was synergic in all ERL-resistant HCC827 cell lines only in RB1.1 low drug concentrations were sufficient to impair cell growth by 70–80%. In all other ERL-resistant lines, in particular in RA2 and RB2, synergy was observed only at high drug concentrations. Remarkably, in RB1.1 cells the majority of experimental points assayed were in the area of the CI plots corresponding to a % of inhibition 70–80% (Fig 7). To validate these data, two other MET inhibitors, PHA and PF, were tested in combination with erlotinib in cell viability inhibition assays. The data essentially similar to the ones obtained with SU and erlotinib confirmed the efficacy of these combination drugs (Table 4).

**Table 4:** Dm_50_ of single agent and drugs combination for parental and ERL-resistant NSCLC cell lines.

	HCC827	RA1	RA2	RB1	RB1.1	RB2
**ERL**	8–30 nM	>10 μM	>10 μM	>10 μM	>10 μM	>10 μM
**SU**	>20 μM	>20 μM	>20 μM	>20 μM	>20 μM	>20 μM
**ERL+SU**	17–150 nM	2.3–4.8 μM	>10 μM	3.5–5.8 μM	220–350 nM	>10 μM
**PHA**	>10 μM	>10 μM	>10 μM	>10 μM	>10 μM	>10 μM
**ERL+PHA**	8–160 nM	2–9 μM	>10 μM	2–8.1 μM	70–170 nM	>10 μM
**PF**	>10 μM	>10 μM	>10 μM	>10 μM	>10 μM	>10 μM
**ERL+PF**	8–45 nM	4.4- >10 μM	>10 μM	>10 μM	30–50 n M	>10 μM

Median effect concentrations (Dm_50_) values are indicated. ERL, SU, PHA and PF inhibitors: Erlotinib, SU11274, PHA-665752, PF-04217903.

Overall the data suggest that *MET* gene amplification and receptor activation are not sufficient to predict efficacy of erlotinib plus MET inhibitor treatment in NSCLC with acquired resistance to erlotinib and that other biomarkers are required for optimal treatment choice.

**Fig 8:**
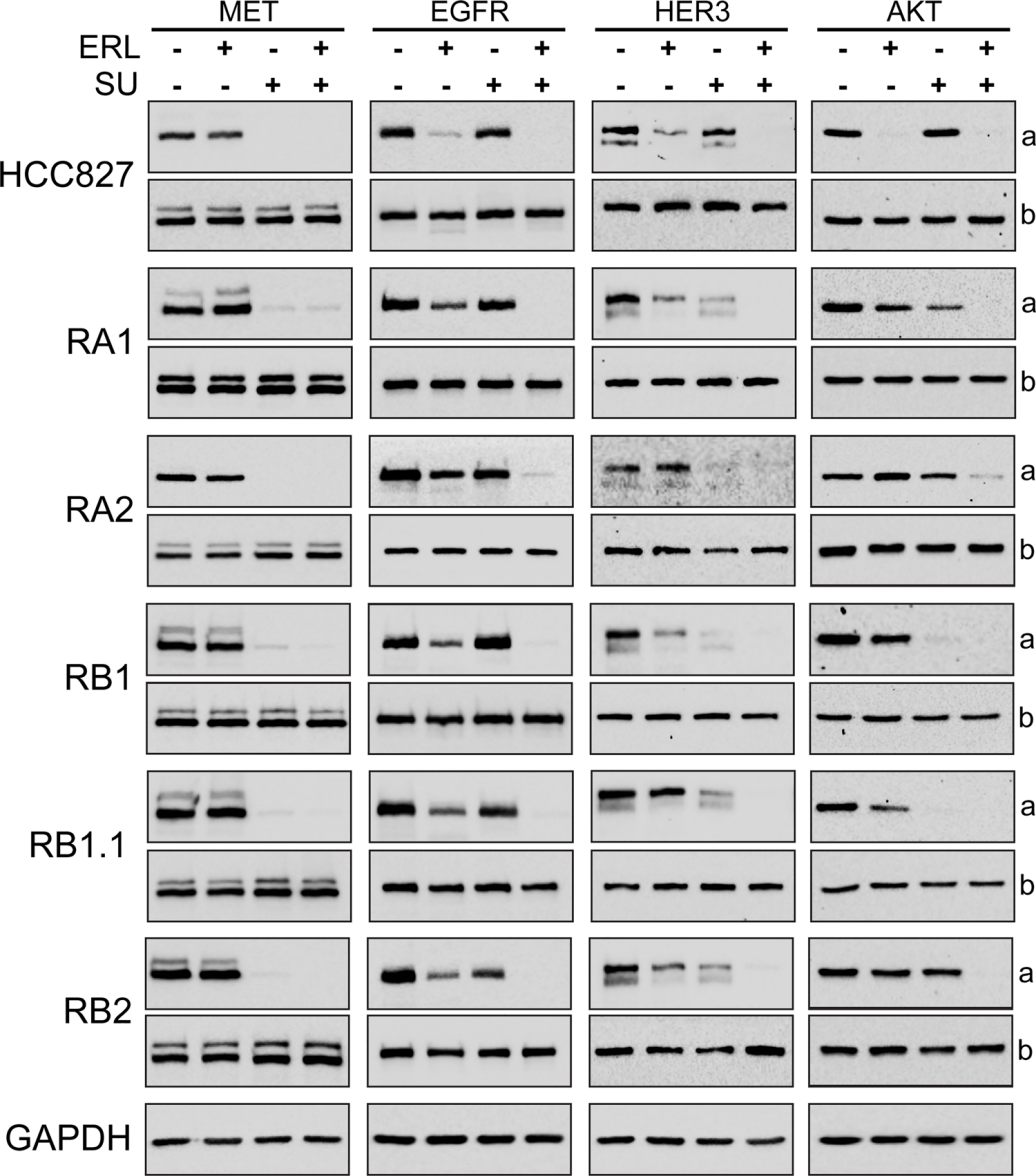
Biochemical analysis of the effects of Erlotinib and MET inhibitors in ERL-resistant NSCLC cell lines. Western blotting of 8% SDS-PAGE of parental and ERL-resistant HCC827 cells treated with ERL (erlotinib, 1 μM), SU (SU11274, 4 μM) for 3 hrs. In (a) are shown representative western blots with antibodies specific for phospho-MET (Y1234/1235), phospho-EGFR (Y1068), phosho-HER3 (Y1289) and phospho-AKT (S473); in (b) western blots for MET, EGFR, HER3 and AKT proteins.

To further investigate the biochemical mechanisms of the combined treatments, the expression of MET, EGFR, HER3 and AKT and their phosphorylation status were investigated by western blotting. In all cell lines, as expected, a strong decrease of MET phosphorylation (Y1234/1235) was observed upon treatment with SU inhibitor or SU in combination with erlotinib (Fig 8). Furthermore, a decrease of pEGFR (Y1068) levels was observed in parental cell line treated with erlotinib and in ERL-resistant cell lines treated with the TKIs combination (Fig 8). These data confirmed the selectivity of SU for MET receptor and indicate that drugs combination could potentially switch off both receptor-mediated signaling pathways. A complete dephosphorylation of AKT (S473) and HER3 was observed in all ERL-resistant cell lines only when the cells were treated with both erlotinib and MET inhibitor (Fig 8). However, a partial reduction of pHER3 levels was observed in the ERL-resistant cell lines treated with SU inhibitor alone indicating likely an effect of SU on HER3-MET heterodimers. In fact MET has been found to form heterodimers with other RTKs, including EGFR, HER2, HER3 and RET [78–80, 26, 31].

In summary these data suggest that this TKIs combination treatment can impair both MET and EGFR signaling pathways but that a strong impact on cell survival can be obtained only in RB1.1 ERL-resistant NSCLC cell line. Ongoing comparative genome hybridization and RNA expression array analyses could be instrumental to identify possible mechanisms leading to differential response to drug treatment combination and/or biomarkers to predict treatment efficacy.

## Conclusion

We have isolated and characterized six ERL-resistant EGFR mutant NSCLC cell lines. We reported alteration in EGFR amplification in one cell line, AXL overexpression and/or MET overexpression and activation in ERL-resistant cell lines, consistently with previous studies indicating that multiple mechanisms may contribute to EGFR TKI treatment resistance. Moreover, our pilot study with EGFR and MET inhibitors demonstrate that *MET* gene amplification and receptor activation are not sufficient to predict a positive effect of drug TKI cocktails on ERL-resistant NSCLCs highlighting the necessity to search for novel biochemical and molecular markers guiding treatment choice in ERL-resistant patients and in ongoing clinical trials.

## Supporting Information

S1 Table Primer nucleotide sequences. (DOCX)
