# Supplementary material for: MET Gene Amplification and MET Receptor Activation Are Not Sufficient to Predict Efficacy of Combined MET and EGFR Inhibitors in EGFR TKI-Resistant NSCLC Cells
Source: PLoS One. 2015 Nov 18;10(11):e0143333. doi: 10.1371/journal.pone.0143333 (PMC4651538; doi:10.1371/journal.pone.0143333)
Supplement: S1 Table — (DOCX) [file pone.0143333.s001.docx]

| **GCN Primers** | **Sequence 5’ > 3’** |
| --- | --- |
| hMETgene F | GCTGGTGGTCCTACCATACATG |
| hMETgene R | CTGGCTTACAGCTAGTTTGCCA |
| hEGFRgene F | GTGCAGATCGCAAAGGTAATCAG |
| hEGFRgene R | GCAGACCGCATGTGAGGAT |
| RnasePgene F | AGATTTGGACCTGCGAGCG |
| RnasePgene R | GAGCGGCTGTCTCCACAAGT |
| **Sequencing Primers** | **Sequence 5’ > 3’** |
| KRAS exon 2 F | TTTGAAATAATTTTTCATATAAAGGTGAG |
| KRAS exon 2 R | TCATGAAAATGGTCAGAGAAACC |
| KRAS exon 3 F | TGTAATAATCCAGACTGTGTTTCTCC |
| KRAS exon 3 R | AGCTTATTATATTCAATTTAAACCCACC |
| EGFR exon 19 F | TGCATCGCTGGTAACATCCA |
| EGFR exon 19 R | TCTAGAGCAGAGCAGCTGC |
| EGFR exon 20 F | CTCAAGATCGCATTCATGCG |
| EGFR exon 20 R | CTCTTGCTATCCCAGGAGC |
| **qPCR Primers** | **Sequence 5’ > 3’** |
| hEGFR F | TAACAAGCTCACGCAGTTGG |
| hEGFR R | CCAAGGACCACCTCACAGTT |
| hERBB2 F | GGTTCACCCACCAGAGTGAT |
| hERBB2 R | GGGATCCCATCGTAAGGTTT |
| hERBB3 F | GTCTGTGTGACCCACTGCAACT |
| hERBB3 R | GGGTGGCAGGAGAAGCATT |
| hERBB4 F | GGCTGCTGAGTTTTCAAGGATG |
| hERBB4 R | GCTTCATACGATCATCACCCTGA |
| hMET F | CAGCTGACTTGCTGAGAGGA |
| hMET R | GAGGTTTATCTTTCGGTGCCCA |
| hAXL F | CACCTCCCTGCAGCTTTCC |
| hAXL R | CTGGGACACGAAGGTCTGATG |
| hVIM F | ACACCCTGCAATCTTTCAGACA |
| hVIM R | GATTCCACTTTGCGTTCAAGGT |
| hCDH1 F | ATTCTGATTCTGCTGCTCTTG |
| hCDH1 R | AGTCCTGGTCCTCTTCTC |
| hRPL31 F | CATCCATGGAGTGGGCTTCA |
| hRPl31 R | AGCTTTGTTGAGCCTGGTGT |

S1 Table: Primer nucleotide sequences

GCN, F, R indicate Gene Copy Number, Forward, Reverse.
